# Composite end points and competing risks analysis

**DOI:** 10.1093/icvts/ivae126

**Published:** 2024-07-02

**Authors:** Victor Dayan, Stuart W Grant, James M Brophy, Fabio Barili, Nick Freemantle

**Affiliations:** Centro Cardiovascular Universitario, Cardiac Surgery Department, Hospital de Clinicas, Universidad de la Republica, Montevideo, Uruguay; Division of Cardiovascular Sciences, University of Manchester, ERC, Manchester University Hospitals Foundation Trust, Manchester, UK; McGill University Health Centre, Cardiology Department, Montréal, QC, Canada; Department of Cardiac Surgery, Santa Croce Hospital, Cuneo, Italy; Institute for Clinical Trials, University College London, London, UK

**Keywords:** Composite outcomes, Competing risks, Statistics

## Abstract

Composite end points are common primary outcomes in clinical trials. Their main benefit of utilizing a composite outcome is increasing the number of primary outcome events, meaning fewer participants are required to deliver an adequately powered trial. By combining multiple important end points in the primary outcome rather than having to select only 1, composite end points potentially make clinically meaningful benefits easier to detect and avoid ranking outcomes hierarchically. However, there are a number of important considerations when designing and interpreting clinical trials that utilize composite end points. In this Statistical Primer, issues with composite end points such as competing events, halo effect, risk of bias, time-to-event limitations and the win ratio are discussed in the context of real world clinical trials.

## INTRODUCTION

A composite end point includes 2 or more different individual end points that are combined so that the occurrence of any of the individual end points results in the occurrence of the composite end point. Understanding how they are selected, constructed as well as their benefits and limitations are important for understanding the effect of the evaluated treatment. Through the example of several contemporary cardiovascular (CV) randomized clinical trials, the objective of this statistical primer is to provide an accessible summary of their identification, management and interpretation.

## EVENTS AND SAMPLE SIZE IN CLINICAL TRIALS

Improvements in medical care have decreased the frequency of major events such as myocardial infarction or death in both clinical practice and trials [1]. This improvement in baseline clinical outcomes means demonstrating the benefits of new treatments has become more challenging. Before the era of coronary reperfusion, hospital mortality after an ST-elevation myocardial infarction was 13% (control group of the 1986 GISSI-1 trial) [2]. Consequently, demonstrating a 30% relative reduction mortality with 90% power and a one-sided 2.5% type I (alpha) error (assuming a control group incidence of 13%) would require 3028 patients with 338 events. If for some reason, we were not able to do the trial at that time, and new data published in 1993 demonstrated that the use of tissue plasminogen activator (tPA) had reduced the mortality after ST-elevation myocardial infarction to 6.3% [3], to be able to demonstrate the same relative reduction of 30% mortality with treatment A, we would now need to recruit 6276 patients (again experiencing 338 events). Full details on sample size calculations are discussed in a previous statistical Primer published in this series (https://academic.oup.com/ejcts/article/54/1/4/4994996?login=false).

In survival analysis, the number of events to achieve a specific hazard reduction at a specified power [1-type II (or beta) error] is constant, and so with a lower incidence in the control group, correspondingly increasing numbers of subjects will be required or the time of follow-up will need to be increased. Mortality is an important outcome, and if instead of aiming for a 30% reduction in mortality, we instead aim for a more modest but realistically attainable 10% reduction, with the lower control event rate of 6.3%, the sample size will increase to 63 282 (now requiring 3794 events).

## SELECTING A PRIMARY OUTCOME

The primary outcome of a trial is of utmost importance since it is the outcome on which the overall inference of the trial is based. A useful primary outcome should adequately reflect the disease under consideration, the putative efficacy of the evaluated treatment, be easy to measure, robust to bias, important for the patient and healthcare providers, and evaluable efficiently in a clinical trial.

Clinical outcomes can generally be categorized into objective or subjective outcomes. Objective outcomes are those where there is no minimal uncertainty as to whether the outcome has occurred and are likely to be reliably measured across patient groups, over time, and by different investigators. Mortality is an objective clinical outcome as its definition is universally accepted and other than in exceptional circumstances, it cannot be reasonably influenced by the investigator or patient. For other clinical outcomes that are objective, for example repeat revascularization or readmission, while there is generally high certainty over whether the outcome has occurred, they can potentially be biased by the subjective opinions of either patient or investigator. Some clinical outcomes may appear to be objective, for example myocardial infarction or stroke; however, the occurrence of these outcomes is dependent on outcome definitions, which is not always completely standardized. Also, documentation of the event may be fragmented further complicating adjudication.

Subjective outcomes are based on an individual’s experience and are particularly prone to patient or investigator bias (e.g. NYHA class, or pain) when judged un-blinded to assigned therapy. There is strong empirical evidence that un-blinded outcome assessments lead to a biased overestimated treatment effect [4]. It is also worth noting that this same study showed un-blinded treatment assignment is also associated with an over-exaggeration of the treatment effect.

Objective or subjective outcomes can be selected in isolation as a primary outcome. Alternatively, multiple objective outcomes, multiple subjective outcomes or a combination of both types of outcomes can be combined into a composite end point. Issues with bias and objective outcomes definitions can potentially be compounded when using diverse composite outcomes, so care must be taken and only the most clinically pertinent composite outcomes considered. Finally, for the trial outcome to be important for patients, investigators should consider including objective patient-centred outcomes in the composite outcome [5].

## WHAT IS A COMPOSITE END POINT?

Its outcome is dichotomous, with patients who experience any of the specified individual end points in the composite, considered to have experienced the composite outcome [6]. The normally equal weighting applied to the composite end point may be clinically problematic. For example, a composite outcome, which includes death and rehospitalization, considers rehospitalization as an equal event to death when evaluating the outcome of the composite. Composite end points serve 2 linked purposes: improve our understanding of the efficacy/harm balance of treatment effect and improve the statistical feasibility of clinical trials. Although both purposes are important, the former must prevail. Although there are different types of composite end points, this review will primarily focus on ‘time to first event’ composite outcomes [7].

## ADVANTAGES OF USING COMPOSITE OUTCOMES

Composite outcomes lead to an increase in primary outcome events, which increases statistical power and reduces the risk of type II or ‘false negative’ error [8]. If multiple outcomes are available and they are all assessed independently rather than as a composite, then this introduces multiplicity, which increases the risk of type I error. Composite outcomes overcome the need to have co-primary end points and thus avoids the issue of multiplicity and the consequent need to adjust *P* values. A type I error is the probability of rejecting the null hypothesis given that it is true; in other words, a false-positive finding. Just as the more coin tosses you make, the higher the probability of getting 1 head; the same applies when you test several hypotheses on the same set of data. This can be easily calculated using the binomial probability distribution, P (at least 1 significant result) = 1 – (1–0.05)^k^, where the type 1 error has been set to 0.05 and *k* is the number of tests. Consider you want to evaluate individually 4 different hypotheses in relation to the relative risk of coronary artery bypass grafts (CABG) versus percutaneous coronary intervention (PCI): mortality, stroke, myocardial infarction and rehospitalization simultaneously. In each comparison, you use a nominal significance level of 5%. The overall type I error for the 4 tests will be 19%. This means that you will have a probability of 19% of rejecting 1 or more of the null hypotheses when indeed they are all true [9]. In addition to increasing statistical power, composite outcomes (when carefully defined to include clinical outcomes of similar importance) may decrease censoring and the need of competing risk analysis. Perhaps most importantly, an advantage of composite end points is that when its components do not go in opposite directions (e.g. treatment decreases death and stroke but increases rehospitalization), they offer a global perspective of clinical efficacy.

## DISADVANTAGES OF USING COMPOSITE OUTCOMES

As we will see in the examples below, selecting a composite end point may overestimate the real benefit of a treatment or it may hide serious associated risks. The halo effect is a cognitive bias in which a positive impression (in this case of a treatment benefit based on a composite primary outcome) can influence by how we feel about other aspects of the treatment [10]. In trials in which the composite end point is positive for the evaluated treatment, it may be perceived that it is positive across all the individual components. It is possible that a difference in a subjective outcome at a high risk of bias included in a composite end point can lead to a perceived benefit in other objective clinical outcomes included in the composite (see example 1: TRILUMINATE trial (below).

Ferreira-González *et al.* reported that most composite end points tend to be driven by outcomes considered less important by clinicians and patients [11]. Mortality, present in almost all CV composite end points, often has the lowest event rate and shows the smallest treatment effect, if present at all [11]. Similar inconsistencies in interpretations may also happen in trials with a non-significant composite outcome but with significant differences in objective individual clinical outcomes (see example 2: EXCEL trial).

Although it is not axiomatic, often the most objective outcomes are those that occur least frequently. Specially in open label trials (not placebo or sham controlled trials), the inclusion of less objective, more frequently occurring outcomes in a composite outcome may often exert significant influence on the overall effect. These outcomes may be clinician (ascertainment bias) or patient-driven outcomes (rehospitalization, revascularization, quality of life, etc.). It has been demonstrated that the inclusion of these outcomes was predictive of a statistically significant result for the primary composite outcome [odds ratio 2.24; 95% confidence interval (CI) 1.15–4.34] [7]. Of course, subjective patient-oriented outcomes are immensely important, but their inclusion is best served when evaluated in a blinded manner to avoid any potential of detection bias that might also carry over to influence the primary composite outcome.

## COMPOSITE OUTCOMES AND TIME TO FIRST EVENT ANALYSES

Composite outcomes are often subject to ‘time to first event’ or ‘survival’ analyses where the time to the 1st event is the critical issue in the comparison between both treatments (irrespective of the type of event). Additional events after the initial event or even fatal events occurring after an initial non-fatal event are not considered. Potential issues with composite time to 1st event outcomes are highlighted in the following hypothetical example.

**Figure 1:**
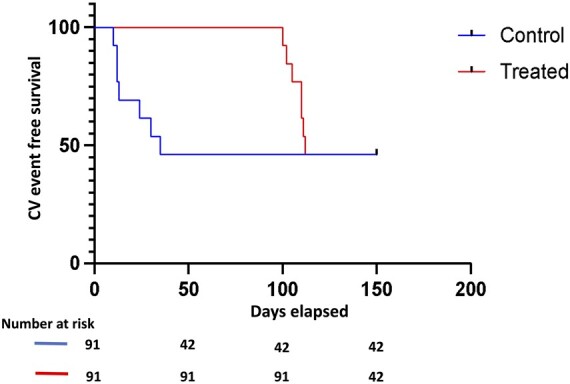
Hypothetical Kaplan–Meier curve. Hospitalization occurred in 49 patients in the control group during the 1st 50 days after randomization and mortality occurred in 49 patients in the treated group after 100 days of randomization. HR = 0.68 (95% CI 0.46–1.02). CV: Cardiovascular.

Imagine conducting a hypothetical trial in which our primary composite outcome is CV events (mortality, stroke, myocardial infarction or hospitalization). Both control and treated groups have 91 patients and, in both groups, the composite outcome occurred in 49 patients. In the treated group, the 49 patients who experienced the outcome all died; in the control group, the 49 events were all hospitalization (Fig. 1).

At 1st glance, one may be tempted to say that the treated group did worse. Nonetheless, since hospitalization occurred earlier during the follow-up, the median time to the composite outcome was in fact shorter for the control group. The hazard ratio for the composite outcome of this hypothetical data is 0.68 (95% CI 0.46–1.02) in favour of the treatment group. This means that treatment reduced the risk for the composite outcome by 32% (even though 49 of their patients died versus no mortality in the control group).

Time-to-event analyses prioritize the time to the event (the shorter the time to the event, the worse for the assigned group) more than absolute number of events or type of event. Consequently, trials that evaluate treatments which decrease risk of short-term events (such as peri-operative myocardial infarction or hospitalization) will have a higher probability of showing beneficial effects with these types of outcomes. In order to improve interpretation of results, reporting of the component-specific analysis should always be performed for time to event composite outcome analyses. To circumvent these limitations, the win ratio has been proposed for the analysis of composite end points [12].

## WIN RATIO APPROACH

The win ratio was 1st proposed in 2012. It is designed to take into account both the clinical importance of the components of composite end points and the relative timing of component events by considering the most important component 1st. Perhaps the most high profile use of the win ratio in CV surgical trials was in the 2nd primary end point 5-year analysis for the low risk PARTNER 3 trial [13, 14].

The win ratio recognizes that patients have different risk profiles and therefore uses risk matched pairs for the analysis (or not matched in case of randomized trials). Patients in each pair are compared hierarchically on who 1st experiences a specific component of the composite end point, if that is not known or neither experienced the component, then the patient who first experienced the next component within the hierarchy of the composite end point is determined.

For example, imagine a trial in which the composite end point is death, stroke or rehospitalization. For each matched pair, we 1st evaluate who had the major event (death) 1st (treatment or control group), if none had the event, then we evaluate the following events in the hierarchical order (stroke and then rehospitalization). If the treatment group had the event later than the control group, it is a win.

Therefore, in the final analysis, you will have 3 types of pairs: winners (in which the patient in the control group had the hierarchical event earlier), losers (in which the patient in the treatment group had the hierarchical event earlier) or tied (in which no event occurred in either patient or an event occurred in 1 patient but follow-up in the other patient is shorter). Win ratio is calculated as the number of winners/losers.

Although this method has some advantages, allows merging more than 1 ‘end-point’ into a single collective analysis that effectively asks ‘did the treatment group patients do better overall than the control group patients?’ and deals with the problem of patients who are missing data on a follow-up assessment due to death, it has several limitations that need to be considered [15].

First, the win ratio does not consider pairs where both patients did not have the event. Therefore, the magnitude of the treatment effect cannot be correctly estimated. Let us say that from 100 pairs (200 patients), 3 are winners (3 control patients had an event) and 2 are losers (2 treatment patients had the event). Relative risk would be 0.67 (treatment reduces 33% the risk for event) and the win ratio will be 1.5 (proportional win rate is 50% greater for a treatment under assessment). The win ratio only tells us about the effect on patients who had the event but tells us nothing about the effect on the overall population (it does not consider tied pairs), in other words the absolute risk reduction or number needed to treat. In the above example, the absolute risk reduction with treatment is 1% (3–2%).

Second even though outcomes are hierarchically ordered, each event may not carry the same clinical weight. A win pair for mortality should not have the same impact as a loser pair for rehospitalization (considering the individual only suffers 1 of the mentioned events). Last, in cases in which ties are frequent, the WIN RATIO may overestimate the treatment effect as shown in the TRILUMINATE example below. There are also important analytical considerations with the use of the win ratio, particularly with regards to matching. Matching relies on multiple factors and calculations. Inaccuracies in matching can arise for a number of reasons including incomplete data, incorrect assumptions or the omission of relevant variables that influence patient outcomes. It is imperative therefore that care is taken when undertaking matching and ensure that methodology is transparently reported and that potential sources of error are minimized.

## DIRECTION OF EFFECT IN EACH INDIVIDUAL COMPONENTS

Each individual component of the composite end point should be consistently associated with the hypothesis assessed within the trial [8]. Including efficacy and safety outcomes within the same composite end point has the potential to lead to individual outcomes within the composite end point having effects in different directions. This makes interpretation of the composite end point problematic. In the SYNTAX (Synergy between PCI with Taxus and Cardiac Surgery) trial [16], the primary outcome was a composite of major adverse cardiac and cerebrovascular events (i.e. death from any cause, stroke, myocardial infarction or repeat revascularization). Although the incidence of major adverse cardiac or cerebrovascular events was lower at 1 year (12.4%) in the CABG group than in the PCI group (17.8%, *P* = 0.002), stroke (one of the individual outcomes of the composite) was significantly lower with PCI (relative risk 0.25; 95% CI 0.09–0.67) and repeat revascularization was significantly higher with PCI (RR 2.29; 95% CI 1.67–3.14). Assuming CABG to be superior to PCI in a composite outcome when in fact it is inferior to one of its individual outcomes (which may be of high importance to individual patients) can be problematic.

Although not the case in the SYNTAX trial at 1 year, components of a composite end point with effects in different directions can potentially neutralize each other. Let us imagine a trial of antibiotics in intensive care unit (ICU) patients. The composite outcome is mortality, ventilator-associated pneumonia and days in the ICU. This antibiotic is very effective and decreases ventilator-associated pneumonia and days in the ICU at the expense of high renal toxicity and overall mortality. Therefore, we find ourselves with individual outcomes that are positive for the evaluated treatment (ventilator-associated pneumonia and days in ICU) and other individual outcomes of the composite which are negative (renal toxicity and mortality). The overall composite may be neutral (no difference between treatment arms) and an incorrect conclusion may be derived regarding both the efficacy and safety of the new treatment [17].

### Competing risks

Competing risks are events that preclude the possibility of the participant experiencing other clinical events of interest. Although they can be relevant for non-composite end points, they are particularly relevant in the context of composite outcomes [18]. In a trial with a CV composite end point, if a patient dies from non-CV causes such as neoplasia, they will not be able to experience the composite CV end point and therefore non-CV death is a competing event. When composite outcomes do not consider competing events, the event rate of the composite will depend on the rates of other competing events. It is therefore important to consider the potential relationship between competing risks and components of the composite outcome.

## SURVIVAL ANALYSIS AND CENSORING

The Kaplan–Meier (KM) method for survival analysis assumes that everyone who enters the study provides 2 important pieces of information: event (yes or no) and follow-up time. Those individuals who did not suffer the event of interest are said to be censored. These patients did not have the event broadly due to 3 reasons: were lost to follow-up, did not have the event by the end of the follow-up period or had another event that precludes the possibility of having the event of interest (competing event). Therefore, a patient who suffers a competing event is censored during survival analysis based on the standard KM method [19].

One of the main principles of censoring in KM survival analysis is the assumption that all censored patients at a specific time have the same probability of suffering the event (non-informative censoring) [19]. A patient who had a competing event (e.g. non-CV death) may violate the non-informative censoring assumption since it is unknown if they had not experienced the competing event if they would have had the same probability of suffering the event of interest (CV death) as those uncensored subjects. Censoring decreases the number of patients who are at risk and therefore, as the denominator decreases (survival is a function of the ratio between patients with the event and patients at risk), the probability of survival is lower (or risk of even is higher). The relative impact of an event is higher the lower the denominator.

In Tables 1 and 2, an example of cumulative survival in a situation without and with censoring is presented. In this example, the event of interest is CV mortality, and non-CV mortality is a competing event. When no censoring occurs (Table 1), freedom from CV mortality at 5 years is 79% (event incidence of 21%); when competing events occur (censoring) (Table 2), freedom from CV-mortality at 5 years is under-estimated, 71% (or it’s complement, event incidence is overestimated, 29%). Therefore, the higher the competing events, the more overestimated the incidence of the event of interest will be.

**Table 1:** Kaplan–Meier method for survival calculation of a situation without censoring.

Year	Number at risk	CV mortality	Censoring	Probability	Cumulative probability
1	100	3	0	0.97 (97/100)	0.97
2	97	5	0	0.95 (92/97)	0.92
3	92	4	0	0.96 (88/92)	0.88
4	88	3	0	0.97 (85/88)	0.85
5	85	6	0	0.93 (79/85)	0.79

In situation A, survival at 5 years is 79% (incidence of event 21%).

CV: Cardiovascular.

**Table 2:** Kaplan–Meier method for survival calculation of a situation with censoring.

Year	Number at risk	CV mortality	Censoring	Probability of event	Cumulative probability
1	100	3	0	0.97 (97/100)	0.97
2	87	5	10	0.94 (82/87)	0.91
3	72	4	10	0.94 (68/72)	0.86
4	58	3	10	0.95 (55/58)	0.82
5	45	6	10	0.87 (39/45)	0.71

In situation B, survival at 5 years is 71% (incidence of event 29%).

CV: Cardiovascular.

## HOW TO DEAL WITH COMPETING RISKS?

Competing events where relevant to the hypothesis of the trial can be included in the composite end point but each individual component of the composite end point should also be analysed separately so that the competing risks can be appropriately highlighted. Competing events that are not included in the composite outcome should also be reported and analysed. This is the preferred approach, but an alternative is to calculate the cumulative incidence function. In contrast to the complement of KM survival (1-KM), cumulative incidence function allows for estimation of the incidence of the occurrence of an event while taking competing events into account [19]. Its calculation is beyond the scope of the current review but it may be noted that it involves a two-step process: calculation of overall survival at each time point (in which all competing events are considered as events) (A) and calculation of probability of failure of the event of interest at each time point (B).

In standard survival analysis, differences in survival between 2 groups are assessed using the log-rank test. In the presence of competing risks, due to the reasons highlighted before, Gray’s test, which is an adaptation of the log-rank test for competing risk data, may instead be used [19]. For Cox proportional hazards when competing events are present, the effect of covariates can be determined using 2 different hazard functions: cause specific (which evaluates the instantaneous hazard in event free individuals) and subdistribution hazard model (Fine-Gray) (which evaluates hazard in event free individuals and those with the competing event). Both models are complementary and may be helpful. Cause-specific hazard models may be more appropriate for addressing epidemiological questions of aetiology (considering it denotes the rate of the primary outcome in individuals who are event free) rather than outcomes in clinical trials, while subdistribution hazard may be of greater interest if one is interested in the overall impact of covariates on the incidence of the outcome of interest [19]. Survival analyses in the presence of competing risks are complex and advice should be sought from an experienced medical statistician. Further information on handling competing risks is also provided in a previous Statistical Primer on advanced survival analyses [20].

## EXAMPLES

### Example 1: TRILUMINATE trial

In the TRILUMINATE Pivotal trial, the authors aimed to evaluate percutaneous tricuspid valve transcatheter edge-to-edge repair among patients with severe tricuspid regurgitation [21]. The primary outcome was a hierarchical composite of death, surgery for tricuspid regurgitation, hospitalization for heart failure and improvement in quality of life, which was analysed using the WIN RATIO. The composite was significant (win ratio 1.48; 95% confidence interval 1.06–2.13; *P* = 0.02) in favour of the transcatheter edge-to-edge repair group.

When we examine the individual outcomes, the difference in composite outcome was primarily driven by the quality of life outcome component with no difference in mortality or heart failure hospitalization. Although clearly important for patients, quality of life outcomes are subjective and potentially open to bias when elicited in an open study. The 6-min walk test, which is a more reliable, and objective outcome associated with QoL also showed no difference between groups. Analysis in the TRILUMINATE trial was performed using the WIN RATIO. The WIN RATIO was 1.48 (95% CI 1.06–2.13). Ties were present in 40% of the pairs. In these cases, Brunner *et al.* [22] propose using win odds in which half of ties are added to the wins and half to the losses treatment group. If win odds is used in TRILUMINATE, the ratio would reduce to 1.28 [15].

### Example 2: EXCEL trial

In the 5-year evaluation of the EXCEL trial, investigators evaluated PCI versus CABG for left main disease on the composite outcome: death, stroke and myocardial infarction [23]. The conclusion was the following: ‘In patients with left main coronary artery disease of low or intermediate anatomical complexity, there was no significant difference between PCI and CABG with respect to the rate of the composite outcome of death, stroke, or myocardial infarction at 5 years’. Although the composite outcome was not significantly different between PCI and CABG, mortality was nominally significantly higher with PCI (odds ratio 1.38; 95% CI 1.03–1.85).

## CONCLUSIONS AND FUTURE DIRECTIONS

Composite outcomes have some advantages and are a good tool to circumvent some of the feasibility issues with clinical trials. However, as with all tools, the benefit of utilizing a composite outcome depends on an understanding of the underlying principles coupled with thoughtful clinical insights about the research question. Careful analysis and interpretation of individual components of the composite end point are mandatory to aid in the interpretation of the real treatment effect. Composite outcomes will inevitably continue to be an important tool in clinical trials. A thorough and balanced understanding of the potential issues with using only composite outcomes to inform guidelines and treatments is essential for both investigators and clinicians. Drug and device approval agencies should stress the inclusion of objective composite outcomes in open label trials. If subjective patient-reported outcomes are included in a composite end point for interventional open label trials (such as quality of life in the RECHARGE trial), then it is vital to ensure that potential sources of bias are minimized as much as possible as composite primary end points can potentially be corrupted when subjective outcomes are included.

## Data Availability

No data has been used for the current manuscript.
